# Structural Studies of a Four-MBT Repeat Protein MBTD1

**DOI:** 10.1371/journal.pone.0007274

**Published:** 2009-10-20

**Authors:** Jitka Eryilmaz, Patricia Pan, Maria F. Amaya, Abdellah Allali-Hassani, Aiping Dong, Melanie A. Adams-Cioaba, Farrell MacKenzie, Masoud Vedadi, Jinrong Min

**Affiliations:** 1 Structural Genomics Consortium, University of Toronto, Toronto, Ontario, Canada; 2 Department of Physiology, University of Toronto, Toronto, Ontario, Canada; University of Cambridge, United Kingdom

## Abstract

**Background:**

The Polycomb group (PcG) of proteins is a family of important developmental regulators. The respective members function as large protein complexes involved in establishment and maintenance of transcriptional repression of developmental control genes. MBTD1, Malignant Brain Tumor domain-containing protein 1, is one such PcG protein. MBTD1 contains four MBT repeats.

**Methodology/Principal Findings:**

We have determined the crystal structure of MBTD1 (residues 130–566aa covering the 4 MBT repeats) at 2.5 Å resolution by X-ray crystallography. The crystal structure of MBTD1 reveals its similarity to another four-MBT-repeat protein L3MBTL2, which binds lower methylated lysine histones. Fluorescence polarization experiments confirmed that MBTD1 preferentially binds mono- and di-methyllysine histone peptides, like L3MBTL1 and L3MBTL2. All known MBT-peptide complex structures characterized to date do not exhibit strong histone peptide sequence selectivity, and use a “cavity insertion recognition mode” to recognize the methylated lysine with the deeply buried methyl-lysine forming extensive interactions with the protein while the peptide residues flanking methyl-lysine forming very few contacts [1]. Nevertheless, our mutagenesis data based on L3MBTL1 suggested that the histone peptides could not bind to MBT repeats in any orientation.

**Conclusions:**

The four MBT repeats in MBTD1 exhibits an asymmetric rhomboid architecture. Like other MBT repeat proteins characterized so far, MBTD1 binds mono- or dimethylated lysine histones through one of its four MBT repeats utilizing a semi-aromatic cage.

**Enhanced version:**

**This article can also be viewed as an enhanced version in which the text of the article is integrated with interactive 3D representations and animated transitions. Please note that a web plugin is required to access this enhanced functionality. Instructions for the installation and use of the web plugin are available in Text S1.**

## Introduction

The nucleosome is the fundamental repeating unit of chromatin. The nucleosome core particle consists of approximately 147 base pairs of DNA wrapped around a histone octamer consisting of 2 copies each of the core histones H2A, H2B, H3, and H4. The four core histones are composed of a globular domain and an unstructured tail. The unstructured tails protrude from the nucleosome and are subject to a number of post-translational modifications including acetylation, methylation, phosphorylation and ubiquitylation [2]. Methylation of lysine and arginine residues on the histones is an important regulator of eukaryotic transcription and genome integrity. These post-translational modifications are thought to act as markers that recruit proteins to specific regions of chromatin [3].

The MBT (Malignant Brain Tumor) repeat is a structural motif of ∼100 amino acids that is conserved from *C. elegans* to humans and exists as tandem repeats [4]. In the human genome, there are at least 9 MBT repeat proteins, each containing two, three or four MBT repeats, respectively. The MBT repeat was originally identified in the *Drosophila* tumor-suppressor protein *L(3)MBT* and mutations of *L(3)MBT* gene cause malignant transformations of the optic neuroblasts [5]. Like other members in the ‘Royal Family’ [6], MBT repeat proteins have been shown to recognize methylated lysine residues on histones [7], and functional studies have suggested important connections between MBT domain-containing proteins and the transcriptional state of chromatin regions [8], [9], [10], [11], [12], [13].

Structural studies of MBT repeat proteins show that, unlike chromodomain [14], [15], [16], [17], MBT repeat proteins use a semi-aromatic cage to accommodate the methyllysine [1], [9], [18], [19], [20], [21]. The difference in the residue composition of the binding pocket of the histone code effectors allows them to discriminate between different lysine methylation states [19]. The chromodomain proteins preferentially bind trimethylated lysine histones whereas MBT repeats show specificity towards the lower methylation states of lysine. To gain insight into the conservation of the semiaromatic cage and its function in methyl recognition, we have determined the structure of the 4-MBT repeat domain of human MBTD1 and characterized its binding specificity against different histone peptides.

## Results and Discussion

**Figure 1:**
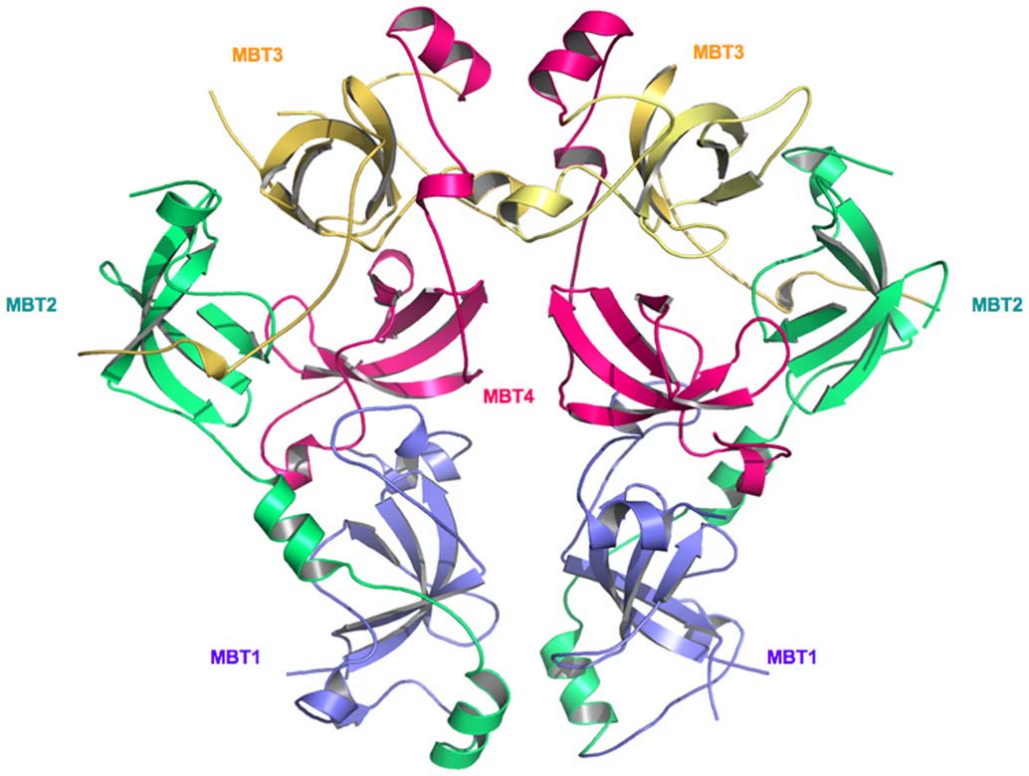
Overall structure of MBTD1. There are two MBTD1 molecules in the asymmetric unit, and each molecule contains four MBT repeats (MBT1, MBT2, MBT3, MBT4), which exhibit irregular rhombus architecture.

**Figure 2:**
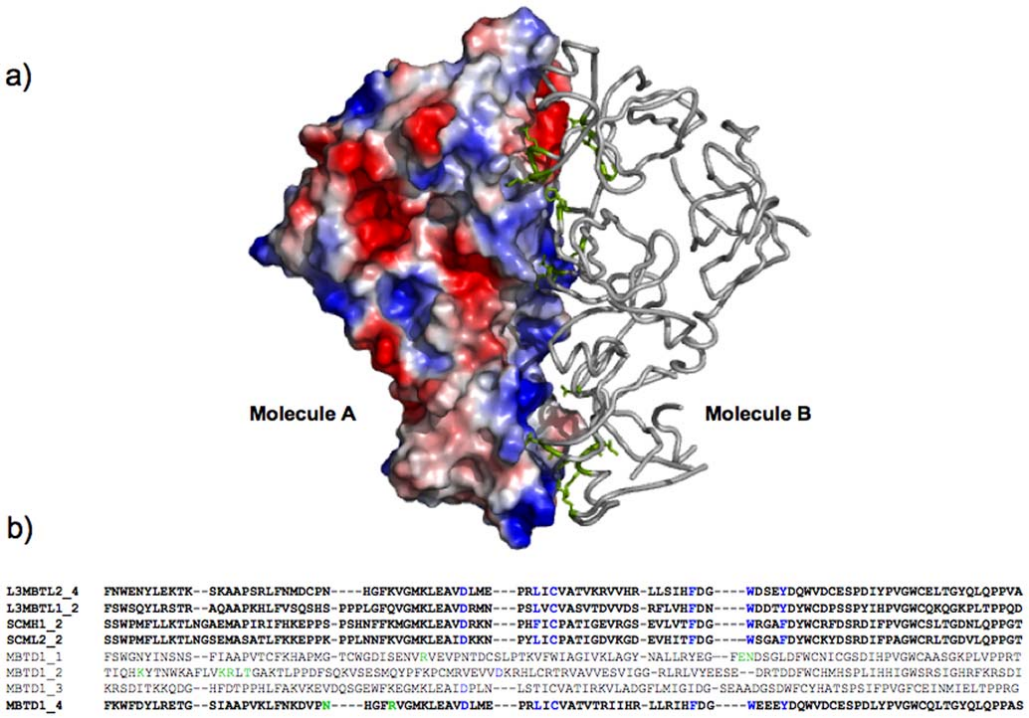
Intermolecular interactions of the two MBTD1 molecules in the asymmetric unit. a) One molecule is shown as surface representation and the other as ribbon representation with residues involved in the intermolecular interactions colored in green. b). Sequence alignment among different MBT modules. All four MBT repeats in MBTD1 were aligned with the methyllysine binding MBT repeats in other MBT proteins (the fourth MBT repeat in L3MBTL2, the second MBT repeat in L3MBTL1, the second MBT in SCMH1 and the second MBT repeat in SCML2). Key residues participating in binding pocket formation are colored in blue and residues involved in the intermolecular interactions colored in green.

MBTD1 is a four MBT repeat protein comprising 628 amino acids. It contains a FCS-type zinc finger at the N-terminus with putative regulatory function [22] and four MBT repeats at the C-terminus. To investigate the crystal structure of the four MBT repeat fragment of MBTD1 and characterize its histone binding specificity, we cloned and purified a human MBTD1 fragment composed of all four MBT repeats (residues 130–566). MBTD1_130–566_ crystallized in the orthorhombic space group P2_1_2_1_2_1_ (a = 70.31 Å, b = 100.90 Å, c = 135.30 Å) with two molecules in the asymmetric unit (Figure 1). Crystal diffraction data and refinement statistics for the MBTD1 structure are displayed in Table 1. In the MBTD1 structure, each molecule contains four MBT repeats that exhibit irregular rhomboid architecture. A narrow channel runs through the middle of the structure and is filled with water molecules. Consistent with previously reported MBT repeat structures, each MBT repeat contains an extended “arm” which packs against a globular β subunit core of the preceding repeat [1]. Structural and sequence alignment show that the β-barrel subunit core region has the highest conservation between MBTD1 and other MBT repeat proteins. The residues involved in intermolecular hydrogen bond interactions between the two molecules in the asymmetric unit are conserved throughout the whole MBT repeat family, possibly implying that MBTD1 functions as a dimer unit (Figure 2). Nonetheless, dynamic light scattering (DLS) experiments and size exclusion experiments demonstrate that MBTD1 is a monomer in solution at the working concentrations (∼5 mg/ml, data not shown). Structurally, the last three MBT repeats in MBTD1 and L3MBTL2 form three-blade propeller architecture. Superposition of the three MBT repeats of L3MBTL1 with those of MBTD1 and L3MBTL2 shows a good structural alignment of Cα positions with a root-mean-squared deviation (RMSD) of 2.345 Å and 2.168 Å, respectively. Furthermore, the MBTD1 can also be well superimposed with L3MBTL2 with a root-mean-squared deviation of 0.697 Å.

**Table 1:** Crystallography data and refinement statistics of MBTD1.

**Data collection**	
Space group	P212121
Cell dimensions	
*a, b, c* (Å)	70.31, 100.90, 135.30
α, β, γ (°)	90.00, 90.00, 90.00
Number of molecules in asu	2
Resolution (Å)	2.5 (2.59–2.50)*
*R_merge_* (%)	9.1 (40.5)
*I/σI*	5.8 (4.1)
Completeness (%)	99.0 (93.1)
Redundancy	6.9 (5.3)
**Refinement**	
Resolution (Å)	2.5
N° reflections	32205
*R_work_/R_free_*	27.0/21.0
N° atoms	
Protein	6457
Water	75
*B*-factors	
Protein	20.2
Water	32.4
R.m.s. deviations	
Bond lengths (Å)	0.02
Bond angles (°)	1.8

**Figure 3:**
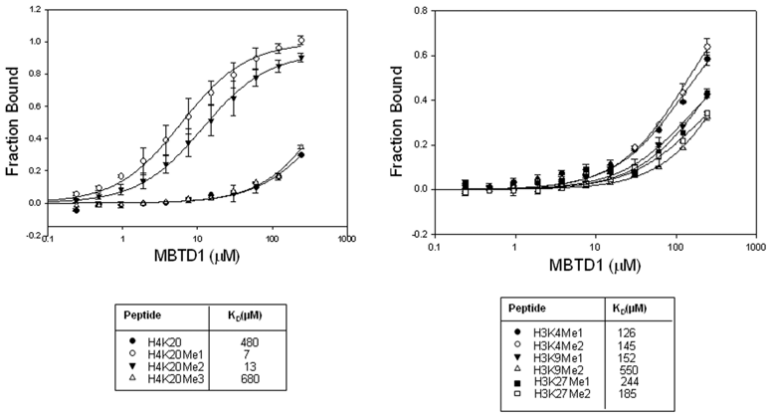
Fluorescence polarization measurements of binding of different histone peptides against MBTD1. The data shows that MBTD1 specifically binds to mono and di-methylated lysine on histone H4K20 and only exhibit negligible binding to the unmodified or trimethylated peptides. In addition, MBTD1 was also shown to bind weakly to mono- or dimethylated lysine histone peptides H3K9, H3K4 and H3K27.

To date, L3MBTL1 [18], [20], [23], L3MBTL2 [1], dScm [9], dSFMBT [13] and SCML2 [21] have been shown to selectively bind lower methylated histone tails. To explore if MBTD1 also possesses histone binding ability, we have carried out the binding studies of the MBTD1 4MBT fragment against a set of fluorescently labeled peptides derived from the N-terminal tails of histones H3 and H4 by means of fluorescence polarization technique (Figure 3). The data revealed that MBTD1 specifically binds to mono and di-methylated lysine on histone H4K20 and exhibit negligible binding to the unmodified or trimethylated peptides. Furthermore, the binding results also show that MBTD1 weakly interacts with mono- or dimethylated lysine histone peptides H3K9, H3K4 and H3K27. This indicates that MBTD1 modestly selectively recognizes lower methylated H4K20 peptide over other histone lysine methylation sites.

**Figure 4:**
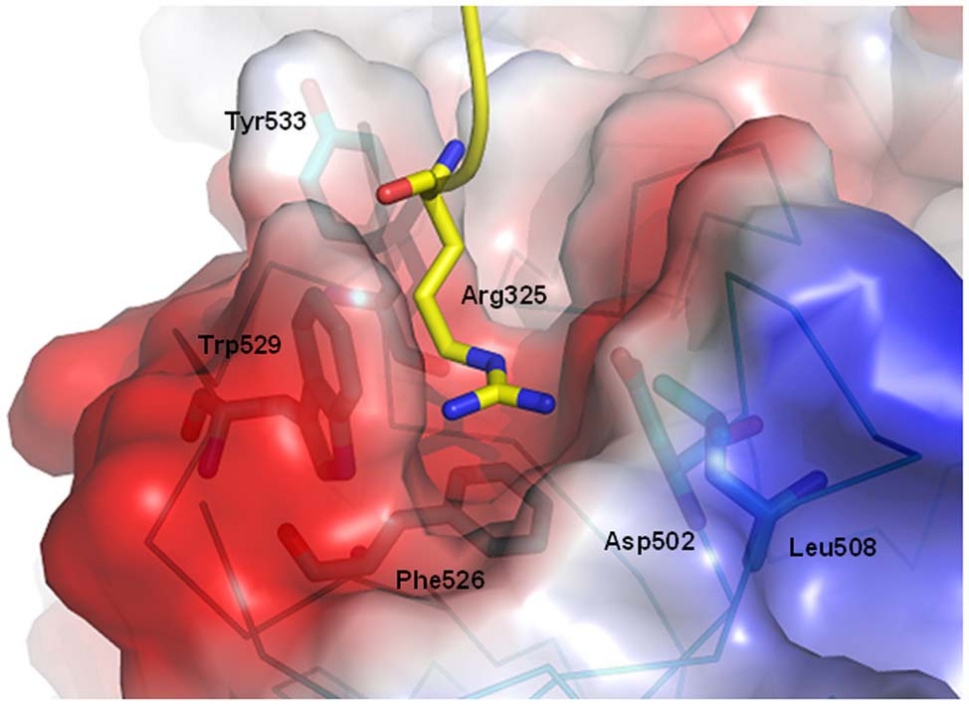
MBTD1 lysine-binding pocket. The MBTD1 binding pocket is formed by aromatic residues Phe526, Trp529, Tyr533, negatively charged Asp502 and Leu508. The binding pocket is occupied by Arg325 from a symmetry related molecule.

Although the four MBT repeats of MBTD1 have similar three-dimensional structures and high sequence identity, only the fourth MBT repeat (MBT4) contains the semi-aromatic cage, which is formed by the loops between the first and second strands and the third and fourth strands of the β- barrel core domain and constitutes the binding site for methyllysine residue, analogous to L3MBTL2. The binding pocket of the MBTD1-MBT4 is shown in Figure 4, where an open cage in MBTD1 is formed by aromatic residues Phe526, Trp529, Tyr533, negatively charged Asp502, and Leu508. Three highly conserved aromatic residues Phe526, Trp529 and Tyr533 form the base and the walls of the hydrophobic pocket. Interestingly, Arg325 from a symmetry related molecule is inserted into the binding pocket, mimicking the methyl-lysine binding in L3MBTL2 [1]. The arginine residue 325 is stabilized by a salt bridge with the pocket residue Asp502, and cation-π and van der Waals and interactions with the aromatic cage residues.

**Figure 5:**
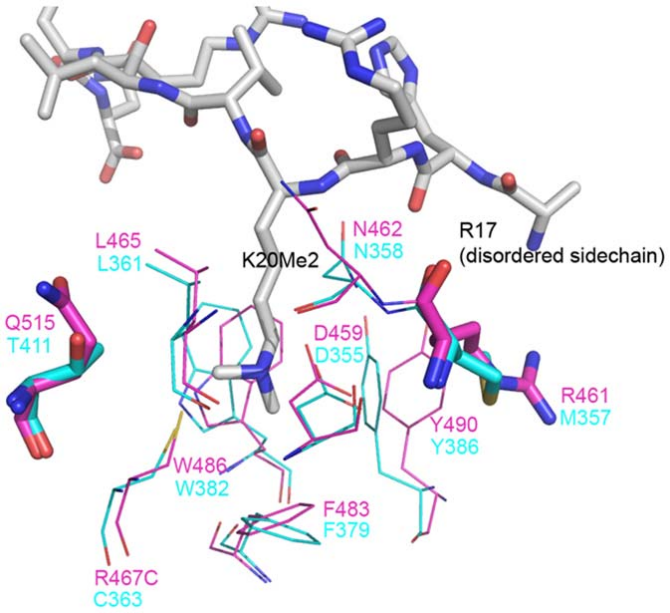
Comparison of MBT3 and MBT2 pockets in L3MBTL1 R467C mutant. MBT3 residues are shown in magenta and MBT2 residues shown in cyan. In the mutant crystal structure Arg461 would potentially clash with Arg17 on the histone peptide and prevent the methylated lysine histone from binding in this pocket.

The utilization of a single MBT repeat for histone binding, despite the structural and sequence similarity shared by all four MBT repeats, is reminiscent of the histone binding mode employed by other MBT containing proteins, including L3MBTL1 [18], [20]. This has been explored in parallel to MBTD1, where only the second MBT domain (MBT2) of the three MBT repeats in L3MBTL1 was bound to histone peptide. Based on sequence and structural alignment, we attributed this phenomenon to the steric hindrance generated by the long or bulky side chain residues (phenylalanine in MBT1 and arginine in MBT3) in the potential pockets instead of cysteine in MBT2. We also suggested that the electrostatic repulsion between the methyllysine and Arginine in MBT3 might be an additional factor, which prevents histone peptide binding. To test our hypothesis and to explore the possibilities of converting a naturally “non-functional” methyllysine binding MBT to a “functional” state, we generated a L3MBTL1 Arg467Cys mutant, which in principle should eliminate both the steric hindrance and electrostatic repulsion in MBT3 based on our prediction. Crystals of the L3MBTL1 R467C mutant were successfully grew in the presence of excess amount of H4K20me2 peptide. However, no electron density was observed in the predicted binding site in the mutated MBT3. ITC experiment of this mutant titrated with H4K20me2 also suggests that there is only one binding site (data not shown). Upon a closer examination of this mutant crystal structure, another residue Arg461 was identified, which potentially clashes with Arg17 on the histone peptide and therefore prevent the methylated lysine histone from binding in this pocket (Figure 5). In MBT2, the same position is occupied by Met357. All known MBT-peptide complex structures characterized to date do not exhibit strong histone peptide sequence selectivity, and use a “cavity insertion recognition mode” to recognize the methylated lysine with the deeply buried methyl-lysine forming extensive interactions with the protein while the peptide residues flanking methyl-lysine forming very few contacts [1]. Although a “functional” MBT3 was not obtained, this mutagenesis study revealed that the histone peptide could not bind to MBT repeats in any orientation.

## Materials and Methods

### Protein expression and purification

The human MBTD1 protein (residues 130–566) was subcloned into pET28a-MHL vector and transformed in *Escherichia coli* BL21 (DE3)-V2R-pRARE2. Cells were grown in Luria-Bertanin media at 37°C until they reach an absorbance at 600nm of approximately 3.0, then cooled down to 14°C followed by induction with 1 mM of isopropyl-β-D-thiogalactoside (IPTG) overnight. Cells were harvested by centrifugation at 7500 rpm for 15 minutes, resuspended in a buffer solution containing 20 mM Tris 8.0, 250 mM NaCl, 10% glycerol and lyzed by sonication. The supernatant fraction obtained by centrifugation at 16,000 rpm for 1 hour and passed through a Ni-NTA Superflow resin (QIAGEN) that had been pre-equilibrated in 20 mM Tris-HCl (pH 8.0), 250 mM NaCl, 10% Glycerol, which was then washed and eluted with 20 mM Tris-HCl (pH 8.0), 250 mM NaCl, 10% Glycerol, 500 mM imidazole. HiTrap Q HP column (GE Healthcare, Piscataway, NJ) and Superdex 75 gel-filtration column (GE Healthcare, Piscataway, NJ) were carried out for further purification. The protein was concentrated to 10 mg/ml in a buffer containing 20 mM Tris-HCl, pH 8.0, 0.2 M NaCl, 10% Glycerol, 1 mM EDTA and 1 mM DTT. The human L3 MBTL1 R467C mutants containing 3 MBT repeats (residues 200–522, 3MBT) were generated by Stratagene's QuikChange method. The mutant protein was expressed and purified as previously described [20]. Fluorescence polarization assays were performed as described in [24].

### Protein crystallization and Structure determination

Crystals of MBTD1 were obtained by macroseeding at 18°C by vapor diffusion of hanging drops of 5 µl of 10 mg ml^−1^ protein solution mixed with 5 µl of a reservoir solution. The reservoir solution contained 20% PEG 3350, 0.2M CaOAc. For cryoprotection, crystals were soaked for a few seconds in a reservoir solution containing 20% (wt/vol) glycerol. The crystals were mounted in a cryoloop and subsequently flash-frozen in liquid nitrogen. X-ray data were collected at 100 K on beamline 23ID-B of Advance Photon Source (APS) at Argonne National Laboratory. A native data set was collected to 2.5 Å resolution. The crystal belongs to space group P212121, with unit cell parameters a = 70.31 Å, b = 100.90 Å, and c = 135.30 Å. There are two molecules in the asymmetric unit that have a VM of 2.42 Å3 Da− 1 and a solvent content of 48.2%. The structure of MBTD1 was determined by molecular replacement using PHASER [25] using the crystal structure of L3MBTL2 (PDB code 3f70) as a search model. Automated building was done with ARP/wARP [26] and manual intervention for corrections. Refinement was carried out using REFMAC [27] in CCP4. The progress of refinement was monitored with Rfree and inspection of 2|Fo|−|Fc| and |Fo|−|Fc| maps in COOT [28]. When Rfree reached 29.8%, TLS refinement was applied and the Rfree dropped to 27.4%. Stereochemical analysis was done with Molprobity. The human L3MBTL1 R467C mutant crystals were grown and its structure was solved using the same methods as we previously reported for their wild-type counterpart [20].

## Supporting Information

Datapack S1 Standalone iSee datapack - contains the enhanced version of this article for use offline. This file can be opened using free software available for download at http://www.molsoft.com/icm_browser.html.
(ICB)

Text S1 Instructions for installation and use of the required web plugin (to access the online enhanced version of this article).
(PDF)
